# Supplementary figures and images for: Subtilisin of Leishmania amazonensis as Potential Druggable Target: Subcellular Localization, In Vitro Leishmanicidal Activity and Molecular Docking of PF-429242, a Subtilisin Inhibitor
Source: Curr Issues Mol Biol. 2022 May 9;44(5):2089–106. doi: 10.3390/cimb44050141 (PMC9164065; doi:10.3390/cimb44050141)

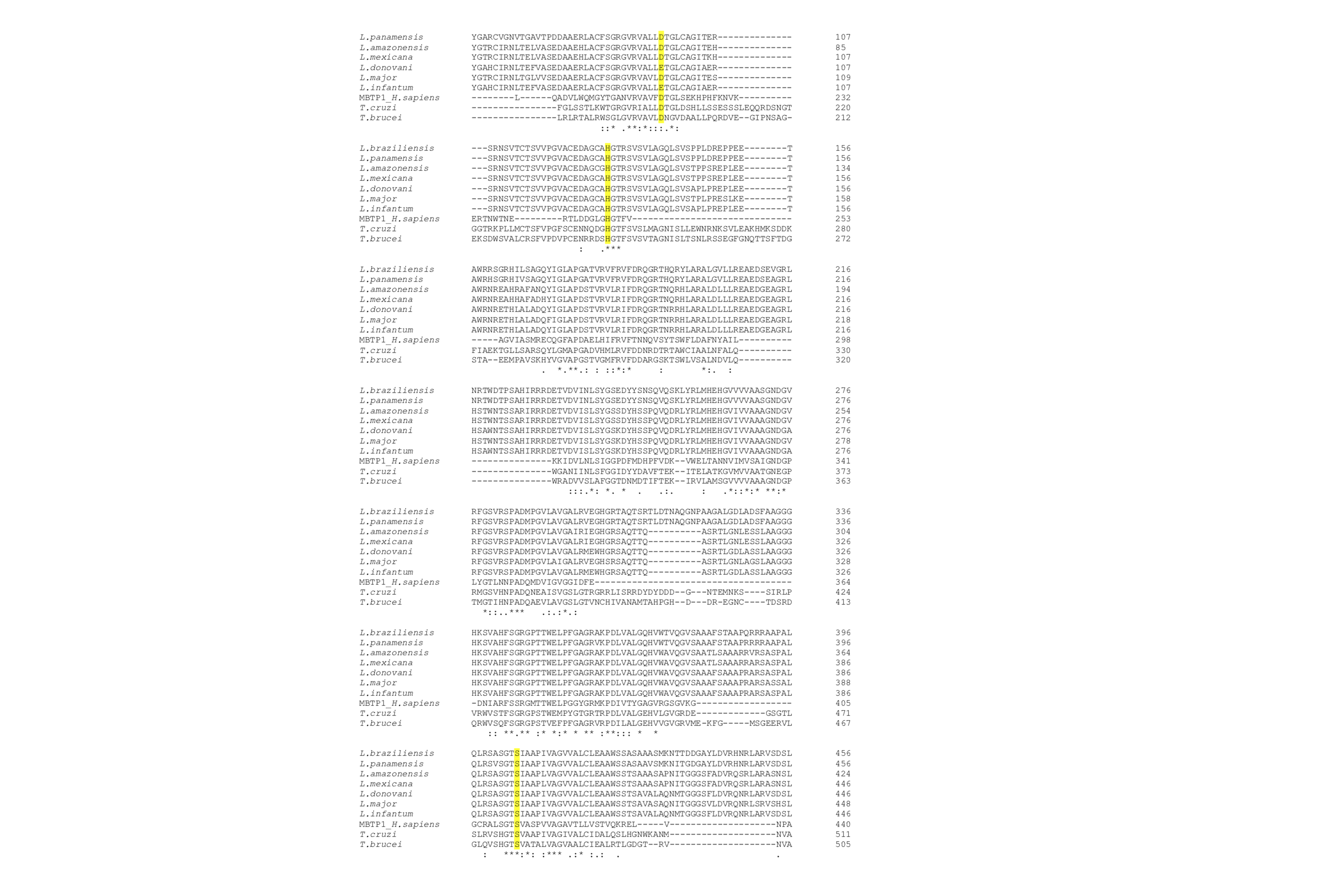

Supplement: Supplementary file 1 [file cimb-44-00141-s001.zip › Suplementares/S1.1.tif]

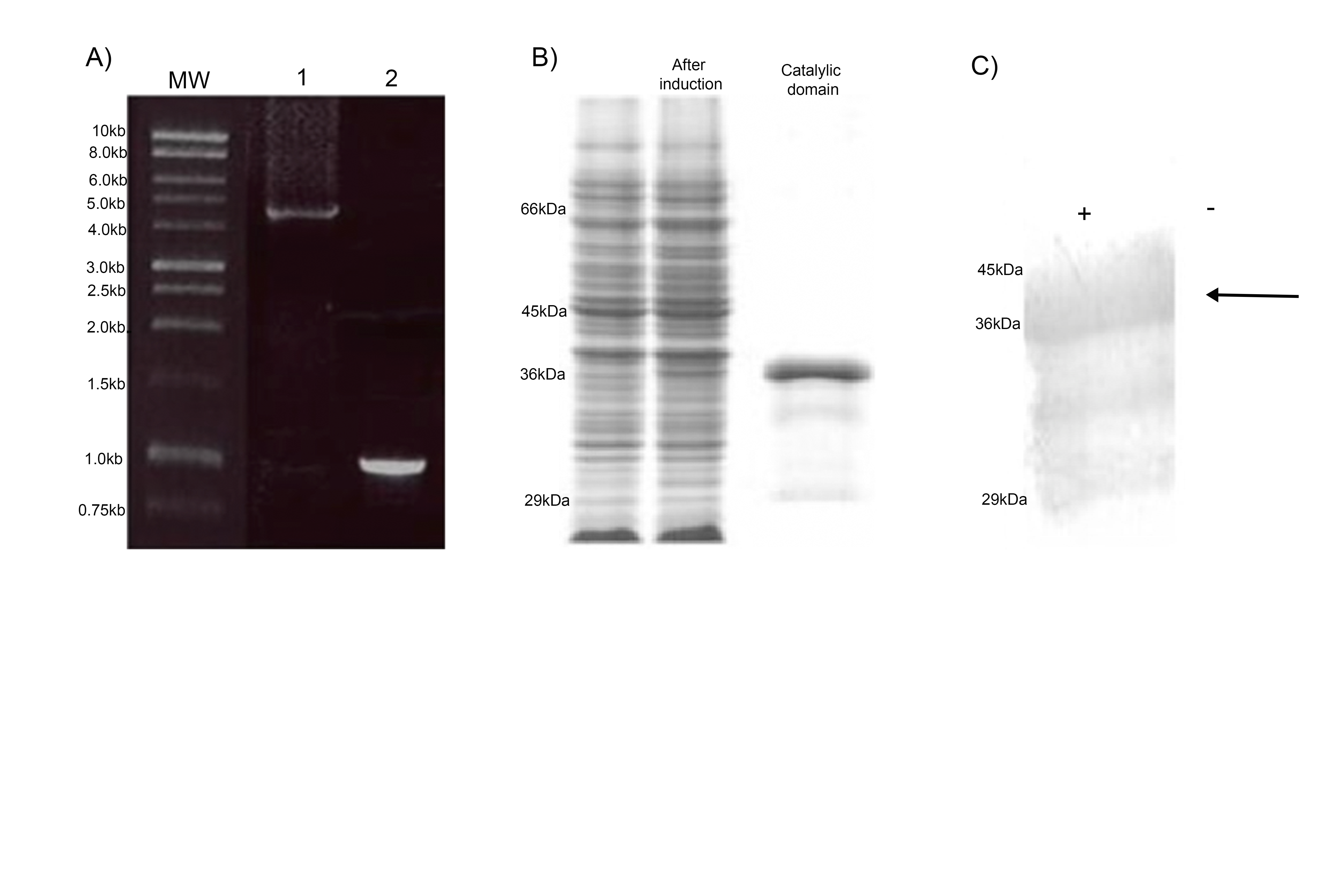

Supplement: Supplementary file 1 [file cimb-44-00141-s001.zip › Suplementares/S2.tif]

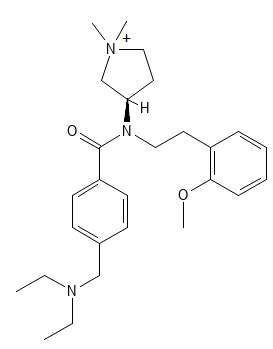

Supplement: Supplementary file 1 [file cimb-44-00141-s001.zip › Suplementares/S4.tiff]

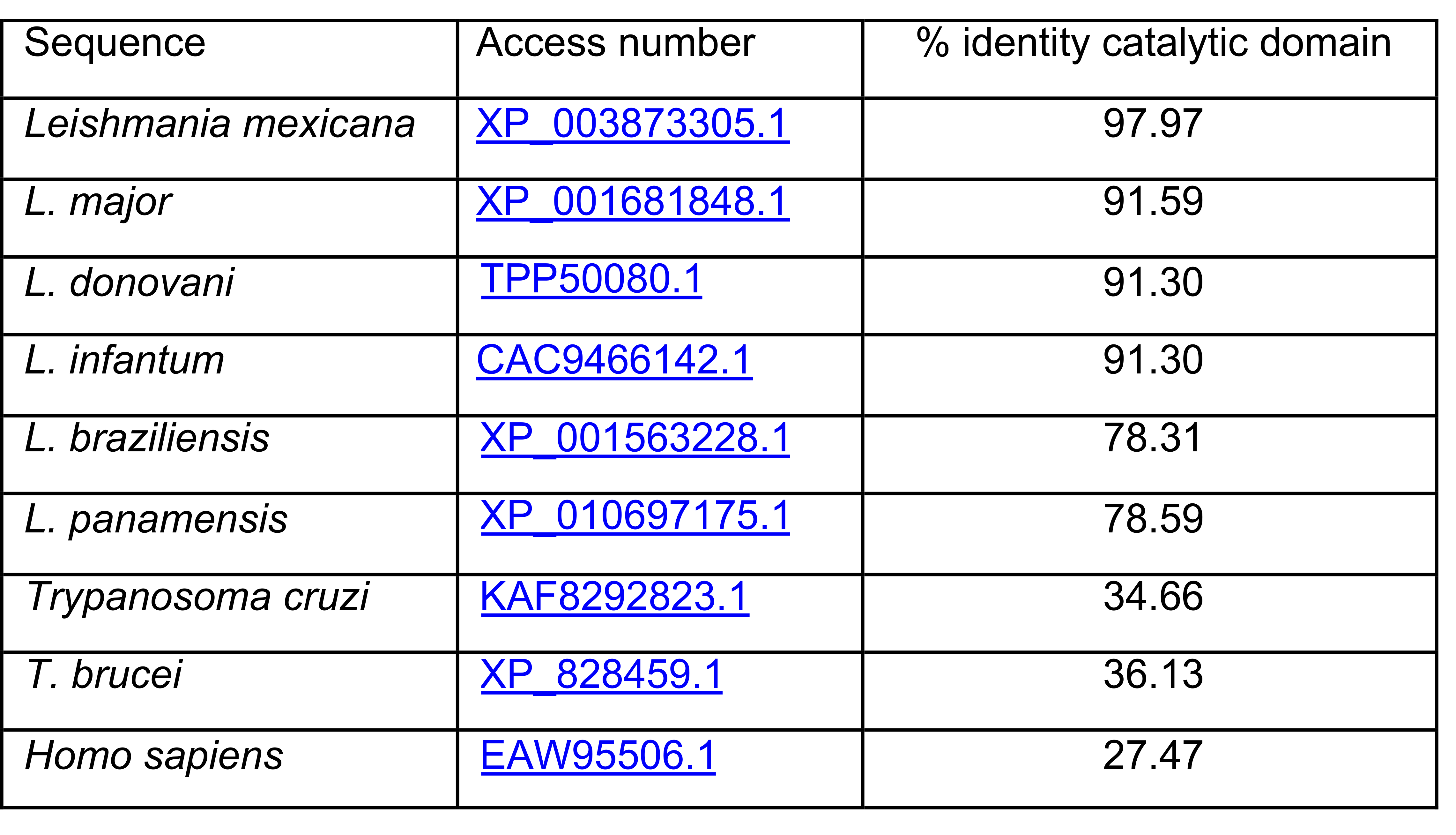

Supplement: Supplementary file 1 [file cimb-44-00141-s001.zip › Suplementares/Tabela S1.tif]
